# Surgical outcomes of atrial cardiac tumors: a single-center 14-case series including right atrial and malignant tumors

**DOI:** 10.1093/jscr/rjaf951

**Published:** 2025-11-25

**Authors:** Masafumi Kudo, Hideki Tsubota, Yuki Akaguma, Masanori Honda, Hitoshi Okabayashi

**Affiliations:** Department of Cardiovascular Surgery, Mitsubishi Kyoto Hospital, 1 Katsuragosho-cho, Nishikyo-ku, Kyoto, 615-8087, Japan; Department of Cardiovascular Surgery, Mitsubishi Kyoto Hospital, 1 Katsuragosho-cho, Nishikyo-ku, Kyoto, 615-8087, Japan; Department of Cardiovascular Surgery, Mitsubishi Kyoto Hospital, 1 Katsuragosho-cho, Nishikyo-ku, Kyoto, 615-8087, Japan; Department of Cardiovascular Surgery, Mitsubishi Kyoto Hospital, 1 Katsuragosho-cho, Nishikyo-ku, Kyoto, 615-8087, Japan; Department of Cardiovascular Surgery, Mitsubishi Kyoto Hospital, 1 Katsuragosho-cho, Nishikyo-ku, Kyoto, 615-8087, Japan

**Keywords:** cardiac tumor, right atrial tumor, left atrial tumor, intimal sarcoma, biphasic synovial sarcoma, autologous pericardial patch

## Abstract

Primary cardiac tumors are rare, with myxomas predominating and usually located in the left atrium. Right atrial tumors are less frequent but clinically significant because of the risks of embolism, hemodynamic compromise, and conduction disturbance. We reviewed 14 patients who underwent surgical resection of atrial tumors at our institution. The cohort included three right atrial and 11 left atrial tumors, with two malignant histologies. All right atrial tumors were resected en bloc with the adjacent wall, requiring autologous pericardial patch closure. Most left atrial tumors also required autologous pericardial patch reconstruction. Malignant tumors determined survival; one patient with intimal sarcoma died early from acute respiratory distress syndrome, and one patient with biphasic synovial sarcoma died 51 months later from pleural dissemination. No recurrence of the benign tumors was observed. Our single-center 14-case series highlights that while the surgical approach varies by tumor location, long-term outcomes are mainly influenced by histology.

## Introduction

Cardiac tumors are rare entities, with primary tumors accounting for <0.3% of all cardiac surgeries [1]. Atrial myxomas represent the majority of these tumors and are predominantly located in the left atrium [2]. Right atrial tumors are much less common but are clinically significant because of their potential to cause pulmonary embolism, right-sided heart failure, or obstruction of venous return [3]. Several studies have reported the general characteristics of cardiac myxomas; however, comparative analyses focusing on right versus left atrial tumors remain limited, especially in studies including malignant histology findings. In this study, we aimed to clarify the clinical features, surgical outcomes, and long-term prognosis of right atrial tumors in comparison with left atrial tumors and contextualize our findings with a review of the relevant literature.

## Case series

The present study included consecutive patients who underwent right atrial tumor resection (RATR) or left atrial tumor resection (LATR) at our institution between June 2009 and May 2023. We compared the different types of tumors, their sizes, sites of attachment, and pre- and postoperative differences between patients who underwent RATR or LATR. Patients in whom an atrial tumor was suspected before surgery but postoperative pathology showed that it was a thrombus were excluded. Patient demographics, clinical characteristics, and perioperative outcomes were retrospectively retrieved from electronic medical records, and all data were anonymized prior to analysis. Patient outcomes were collected through outpatient visits and telephone follow-up. This study was approved by the Institutional Review Board and adhered to the ethical standards of the Declaration of Helsinki. Owing to the retrospective nature of this study, individual patient consent was not obtained. No patient information is included in this manuscript. This study was approved by the Institutional Ethics Committee of our institution (Approval Number: 25-20). Data were collected retrospectively from medical records and analyzed using SPSS version 31 (IBM Corp., Armonk, NY, USA). Continuous variables are expressed as medians with ranges and were compared between groups using the Mann–Whitney *U* test. Categorical variables are presented as numbers and percentages, and intergroup differences were evaluated using Fisher’s exact test. Statistical significance was set at *P* < .05.

**Figure 1:**
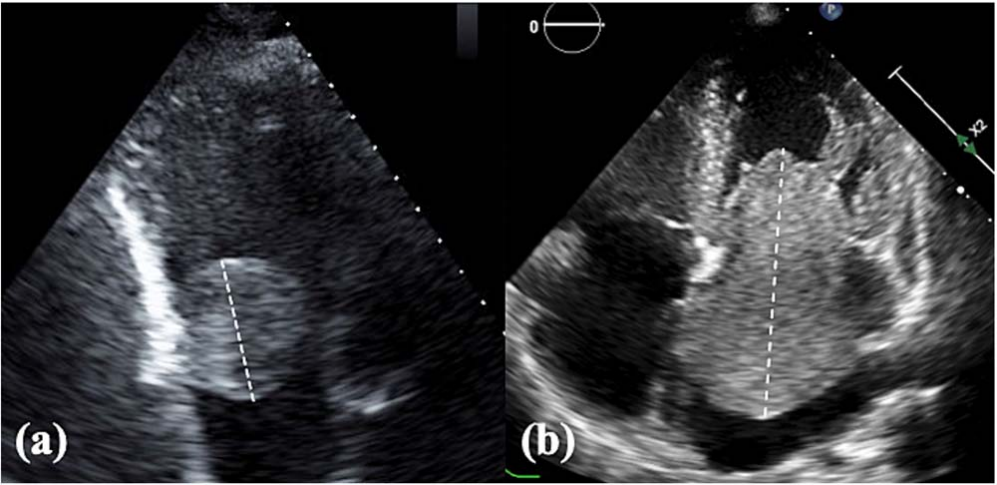
TTE findings. (a) Right atrial tumor and (b) left atrial tumor. Tumor size was assessed using the longest diameter measured by TTE. TTE, transthoracic echocardiography.

A total of 14 patients who underwent atrial tumor resection were included, comprising three (21%) and 11 (79%) patients in the RATR and LATR groups, respectively. The baseline characteristics are presented in Table 1. The median age was 61 years in the RATR group and 68 years in the LATR group, with no significant difference between the two groups (*P* = 1.0). Male sex was more frequent in the RATR group (100% vs. 45%), although the difference was not statistically significant (*P* = .21). There were no significant intergroup differences in body mass index, comorbidities, or the presenting symptoms. One patient in the RATR group presented with complete atrioventricular block complicated by heart failure. In the LATR group, one patient had right hemiparesis preoperatively because of a tumor embolism. Tumor size was measured as the maximum diameter using transthoracic echocardiography (Fig. 1). Tumor size tended to be larger in the LATR group (median, 30 vs. 14 mm); however, this difference was not statistically significant (*P* = .3). The most common site of tumor attachment was the atrial septum in both groups, and there was no significant difference in its distribution (*P* = 1.0). A history of malignant tumors was observed in one patient (gastric cancer) in the RATR group and in three patients (renal cell carcinoma, liposarcoma, and breast cancer) in the LATR group.

**Table 1:** Baseline characteristics of patients with atrial tumors

Variable	RATR (*n* = 3)	LATR (*n* = 11)	*P* value
Age (years), median [range]	61 [46–74]	68 [41–85]	1.000
Male, *n* (%)	3 (100%)	5 (45%)	.209
Height (cm), median [range]	169 [166–177]	155 [142–175]	.400
BMI (kg/m^2^), median [range]	24.3 [19.6–27.3]	22.6 [18.5–27.7]	1.000
LVEF (%), median [range]	66.1 [61.5–85.2]	75 [57.0–83.8]	0.700
Symptoms, *n* (%)	3 (100%)	9 (82%)	1.000
Type of symptoms, *n* (%)			
Pulsation	2 (67%)	6 (55%)	1.000
Paralysis	0	1 (10%)	1.000
Dyspnea	1 (33%)	2 (18%)	1.000
Size of tumor (mm), median [range]	14 [14–36]	30 [12–82]	0.300
Attachment site of tumor, *n* (%)			1.000
Atrial septum	2 (67%)	5 (45%)	1.000
Roof of left atrium	0	2 (18%)	1.000
Posterior wall of left atrium	0	4 (36%)	0.506
Tricuspid valve	1 (33%)	0	0.214
Medical history, *n* (%)			
HT	0	4 (36%)	0.505
HL	1 (33%)	2 (18%)	1.000
DM	1 (33%)	1 (10%)	0.396
CKD (eGFR < 40 ml/min)	0	1 (10%)	1.000
COPD (FEV 1.0% < 70%)	0	2 (18%)	1.000
HD	0	0	-
AF	2 (67%)	3 (27%)	0.505
Smoking, former/current	3 (100%)	4 (36%)	0.192
Malignant tumor	1 (33%)	3 (27%)	1.000

RATR, right atrial tumor resection; LATR, left atrial tumor resection; BMI, body mass index; LVEF, left-ventricular ejection fraction; HT, hypertension; HL, hyperlipidemia; DM, diabetes mellitus; CKD, chronic kidney disease; COPD, chronic obstructive pulmonary disease; HD, hemodialysis; AF, atrial fibrillation.

The operative data are presented in Table 2. Extracorporeal circulation and aortic cross-clamp times were comparable between the groups. The surgical approach differed between the groups: all patients in the RATR group underwent right atriotomy, whereas most patients in the LATR group underwent left atriotomy, showing a trend toward significance (*P* = .055). In all RATR cases, the defect resulting from en bloc resection of the tumor and adjacent atrial wall was closed with autologous pericardial patch repair, whereas most LATR cases (73%) also required autologous pericardial patch reconstruction; this difference was not statistically significant (*P* = 1.0). Concomitant procedures, including valve surgery, coronary artery bypass grafting, and arrhythmia surgery, were performed only in the LATR group, but the differences were not significant.

**Table 2:** Operative data of patients with atrial tumors

Variable	RATR (*n* = 3)	LATR (*n* = 11)	*P* value
ECC time (min), median [range]	150 [129–167]	130 [98–274]	1.000
Cross-clamp time (min), median [range]	100 [61–109]	92 [61–202]	1.000
Approach, *n* (%)			
Right atriotomy	3 (100%)	3 (27%)	.055
Left atriotomy	0	8 (73%)	.055
Closure method of the wall defect, *n* (%)			
Pericardial patch closure	3 (100%)	8 (73%)	1.000
Direct closure	0	1 (10%)	1.000
Concomitant surgical procedure, *n* (%)			
Mitral valve procedure	0	1 (10%)	1.000
Tricuspid valve procedure	1 (33%)	0	.214
CABG	0	2 (18%)	1.000
Arrhythmia procedure	1 (33%)	2 (18%)	1.000

RATR, right atrial tumor resection; LATR, left atrial tumor resection; ECC, extracorporeal circulation; CABG, coronary artery bypass grafting.

**Figure 2:**
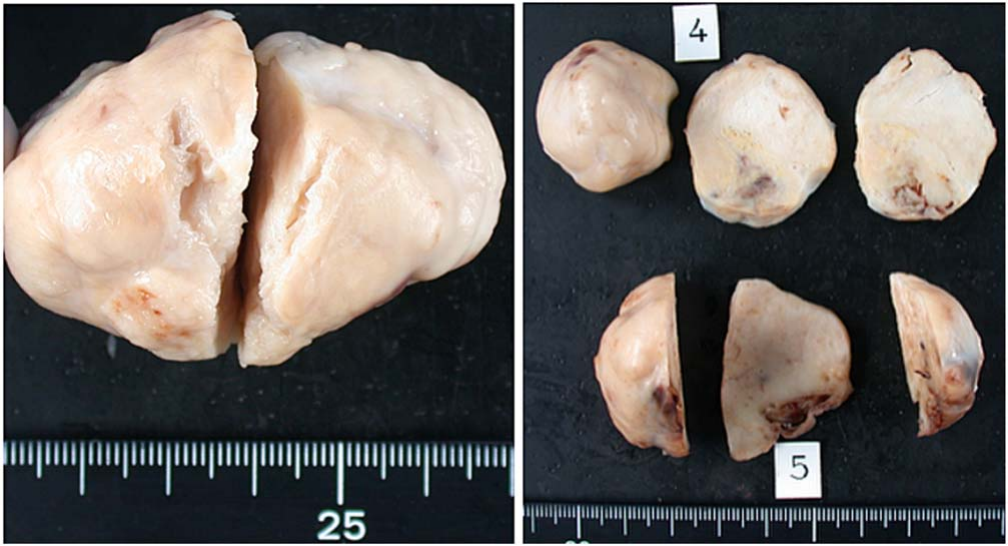
Biphasic synovial sarcoma. Grossly, the tumor appeared as a potato-like mass. The cut surface was slightly mucoid and whitish translucent, and the resection margin showed a finely granular appearance.

**Figure 3:**
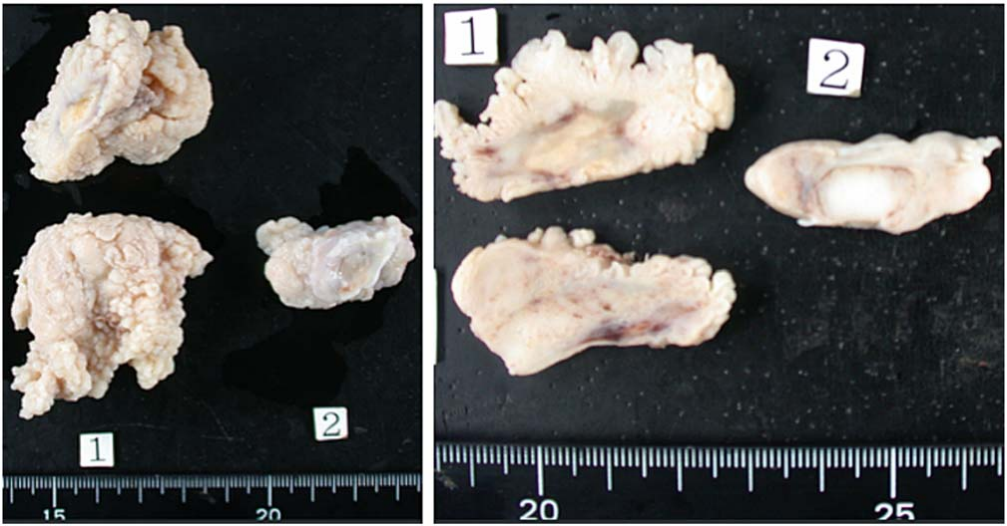
Intimal sarcoma. Grossly, the tumor was a large, cauliflower-like mass with partially cystic areas. It appeared milky white and showed no mucinous features.

The postoperative outcomes are summarized in Table 3. No significant differences were observed in postoperative complications, including cerebral infarction, dialysis, and new-onset atrial fibrillation. Permanent pacemaker implantation was required in two (67%) and three (27%) patients in the RATR and LATR groups, respectively (*P* = .51). The median follow-up duration was 103 months in the RATR group and 158 months in the LATR group, with no significant difference between the groups (*P* = .7). Histopathological diagnosis revealed malignant tumors in one patient in the RATR group (Fig. 2) and one patient in the LATR group (Fig. 3)**,** with no significant between-group difference (Fisher’s exact test, *P* = .396). One patient in the RATR group with biphasic synovial sarcoma died of respiratory failure due to pleural dissemination 51 months after surgery, and one patient in the LATR group with intimal sarcoma died on postoperative day 8 due to acute respiratory distress syndrome (ARDS) caused by pleural dissemination. No cardiac tumor recurrence was observed in either group. Newly developed malignancies after surgery were observed in two patients in the RATR group (lung and prostate cancers) and four patients in the LATR group (ovarian, esophageal, and two liver cancers). Death from malignant tumors occurred in one patient in the RATR group and two patients in the LATR group (*P* = 1.0). Overall survival did not differ significantly between the groups.

**Table 3:** Postoperative outcomes and follow-up

Variable	RATR (*n* = 3)	LATR (*n* = 11)	*P* value
Follow-up (mo), median [range]	103 [51–125]	158 [0.3–194]	.700
Histopathology, *n* (%)			
Myxoma	1 (33%)	10 (90%)	.093
Papillary fibroelastoma	1 (33%)	0	.214
Intimal sarcoma	0	1 (10%)	1.000
Biphasic synovial sarcoma	1 (33%)	0	.214
Postoperative complication, *n* (%)			
Cerebral infarction	0	1 (10%)	1.000
Systemic embolism	0	0	-
Reoperation for bleeding	0	0	-
Postoperative dialysis	0	1 (10%)	1.000
Mediastinitis	0	0	-
POAF	2 (67%)	7 (63%)	1.000
Pacemaker implantation	2 (67%)	3 (27%)	.505
Recurrence of cardiac tumor	0	0	-
Duration of hospital stay (days), median [range]	19 [15–25]	13 [8–51]	.700
30-day readmission	1 (33%)	0	0.214
Death within 30 days	0	1 (10%)	1.000
Death from malignant tumor	1 (33%)	2 (18%)	1.000
Death from any cause	1 (33%)	3 (27%)	1.000

POAF, postoperative atrial fibrillation.

## Discussion

In this single-center retrospective series of 14 patients, the perioperative characteristics and long-term outcomes were broadly comparable between the RATR and LATR groups. The RATR group uniformly required right atriotomy and autologous pericardial patch closure, whereas the LATR group was more often approached via left atriotomy. Histopathology showed similar rates of malignant tumors in both groups (1/3 vs. 1/11; *P* = .396), no cardiac tumor recurrence was observed, and overall survival did not differ. We investigated whether the RATR and LATR groups differed in terms of presentation, surgical management, and outcomes. Our data indicate that the RATR group did not confer worse short- or long-term outcomes compared with the LATR group, nor a higher malignant proportion in this cohort. ElBardissi *et al*. reviewed a 48-year institutional experience of 323 patients with primary cardiac tumors and reported that surgical resection achieved excellent early survival, with long-term outcomes mainly determined by histologic type rather than tumor location. Their study demonstrated that benign tumors, especially myxomas, were associated with a favorable prognosis, whereas malignant tumors had poor outcomes, regardless of the atrial side [4].

Three points merit emphasis. The main practical difference lies in the surgical approach and the method of atrial wall reconstruction. In the RATR group, the tumor was resected en bloc with the involved atrial wall, and the resulting defect was closed with an autologous pericardial patch in all cases. In the LATR group, most patients (73%) required autologous pericardial patch reconstruction, although direct closure was possible in some cases. These differences, rather than adverse events or survival, represent the main practical distinction between the two groups. This observation is consistent with the early reports by Richardson *et al*., who emphasized that atrial myxoma resection frequently results in wall or septal defects requiring patch reconstruction [5]. Similarly, Ryu *et al*. reported that in malignant cardiac tumors, atrial wall defects were reconstructed using autologous pericardium or other patch materials, underscoring the importance of adequate reconstruction strategies, irrespective of tumor histology [6].

Second, although the numbers were small, conduction disturbances requiring permanent pacemaker implantation occurred more frequently in the RATR group (67% vs. 27%; *P* = .51), which was consistent with the proximity of resections to the conduction system. Notably, one patient in the RATR group presented with a complete atrioventricular block preoperatively. Consistent with this finding, Bateman *et al*. reported that among 11 patients undergoing left atrial myxoma resection, early postoperative conduction disturbances were common, and two patients required permanent pacemaker implantation, underscoring the risk of injury to the conduction system during tumor resection [7].

Third, we experienced an early death in an intimal sarcoma case in the LATR group due to ARDS from pleural dissemination, underscoring that outcomes hinge more on histology than on the atrial side. Cardiac intimal sarcoma is an extremely rare and highly aggressive primary malignant tumor with a dismal prognosis, often resulting in death within months despite surgical intervention. A rapid review of reported cases has demonstrated high morbidity and mortality rates, emphasizing the aggressive nature of the neoplasm [8]. Similarly, Nistor *et al*. reported that the median survival after diagnosis ranges from 17 to 24 months, underscoring that long-term survival is uncommon, even with radical treatment [9]. We also encountered a biphasic synovial sarcoma in the right atrium. Cardiac synovial sarcomas are exceedingly rare, with only a few cases reported in the literature, and the biphasic subtype is even less common. These tumors are characterized by aggressive local invasion and a high potential for metastasis, resulting in a poor prognosis despite surgical resection. Our institution has previously reported this case as an individual report [10]. The inclusion of this patient in the present series allowed us to provide extended follow-up data, showing that the patient eventually succumbed to respiratory failure due to pleural dissemination 51 months after surgery. This case highlights the malignant potential of synovial sarcoma and underscores the importance of long-term surveillance in patients with primary cardiac sarcomas.

This study is limited by its retrospective, single-center design, and small sample size, particularly in the RATR group, which reduces the statistical power. Histologic heterogeneity and reliance on echocardiographic measurements for tumor size (which may underestimate dimensions compared with pathology) also constrain interpretation. Selection and information biases cannot be excluded from this study.

In this cohort, the RATR and LATR groups showed similar malignant rates, perioperative complications, and survival, whereas the surgical approach and reconstruction differed by tumor location. These findings support individualized operative planning, anticipating autologous pericardial patch closure, discussing conduction-related risks for the RATR group, and vigilant oncologic assessment whenever malignancy is suspected, regardless of the atrial side.
